# Rational Construction of Honeycomb-like Carbon Network-Encapsulated MoSe_2_ Nanocrystals as Bifunctional Catalysts for Highly Efficient Water Splitting

**DOI:** 10.3390/molecules29163877

**Published:** 2024-08-16

**Authors:** Changjie Ou, Zhongkai Huang, Xiaoyu Yan, Xiangzhong Kong, Xi Chen, Shi Li, Lihua Wang, Zhongmin Wan

**Affiliations:** 1 College of Mechanical Engineering, Hunan Institute of Science and Technology, Yueyang 414006, China; ouchangjie06@163.com (C.O.); 822311110535@vip.hnist.edu.cn (Z.H.); hunanligong4066@163.com (X.Y.); li_shi@csu.edu.cn (S.L.); hnlgwlh@163.com (L.W.); 2 School of Energy and Electrical Engineering, Hunan Institute of Science and Technology, Yueyang 414006, China; xichen2013@hnu.edu.cn

**Keywords:** honeycomb-like network, freeze-drying, MoSe_2_/NC composites, bifunctional electrodes, hydrogen evolution

## Abstract

The scalable fabrication of cost-efficient bifunctional catalysts with enhanced hydrogen evolution reaction (HER) and oxygen evolution reaction (OER) performance plays a significant role in overall water splitting in hydrogen production fields. MoSe_2_ is considered to be one of the most promising candidates because of its low cost and high catalytic activity. Herein, hierarchical nitrogen-doped carbon networks were constructed to enhance the catalytic activity of the MoSe_2_-based materials by scalable free-drying combined with an in situ selenization strategy. The rationally designed carbonaceous network-encapsulated MoSe_2_ composite (MoSe_2_/NC) endows a continuous honeycomb-like structure. When utilized as a bifunctional electrocatalyst for both HER and OER, the MoSe_2_/NC electrode exhibits excellent electrochemical performance. Significantly, the MoSe_2_/NC‖MoSe_2_/NC cells require a mere 1.5 V to reach a current density of 10 mA cm^−2^ for overall water splitting in 1 M KOH. Ex situ characterizations and electrochemical kinetic analysis reveal that the superior catalytic performance of the MoSe_2_/NC composite is mainly attributed to fast electron and ion transportation and good structural stability, which is derived from the abundant active sites and excellent structural flexibility of the honeycomb-like carbon network. This work offers a promising pathway to the scalable fabrication of advanced non-noble bifunctional electrodes for highly efficient hydrogen evolution.

## 1. Introduction

With the rapid expansion of energy demand and serious environmental pollution, clean energy has attracted much attention in recent years. Hydrogen is regarded as one of the most potentially clean energy sources due to its environmental friendliness and abundant sources [1,2,3,4,5,6]. With the advantage of abundant resources, no pollution and nontoxicity, the electrocatalytic pyrolysis of water is the best way to produce hydrogen. However, the hydrogen evolution reaction (HER) during the electrolysis of water usually suffers from several serious drawbacks, such as high energy consumption and low efficiency, which limits its commercial application [7,8,9,10,11]. Therefore, it is necessary to develop catalytic materials to facilitate the HER performance during electrolysis of water. Pt-based materials have the best catalytic performance for HER, but the high cost limits their large-scale application [12,13,14,15,16].

Transition metal selenides (TMS) are considered to be one of the best alternatives because of their excellent electrical conductivity, low ionization energy, low hydrogen adsorption barrier, and high electrochemical activity [17,18,19,20]. Among them, MoSe_2_ has been widely studied by researchers. For example, Setayeshgar et al. prepared MoSe_2_ using Na_2_SeO_3_ and MoCl_5_ as the Se and the Mo sources, respectively [21]. Zhao et al. synthesized the non-defective MoSe_2_ by a hydrothermal method with an overpotential of 364 mV at 10 mA cm^−2^ and Tafel slope of 112 mV dec^−1^ [22]. However, the relatively low intrinsic conductivity and structural stability of pure MoSe_2_ materials usually lead to their unfavorable catalytic properties. Qian et al. reported the fabrication of Zn-doped MoSe_2_ nanosheets synthesized by a one-step hydrothermal method. When tested in 0.5 M H_2_SO_4_, the Zn-doped MoSe_2_ had a favorable catalytic performance [23,24]. The purpose of full water electrolysis is to evaluate the efficiency and stability of the catalyst in the actual water electrolysis process and its performance in long-term operations. This is crucial to the development of efficient and low-cost water electrolytic hydrogen production technology, especially in promoting the wide application of hydrogen energy as a clean energy source.

Many strategies have been developed to solve the above-mentioned disadvantages of MoSe_2_-based materials, such as doping, carbon coating and constructing porous structures [25,26,27,28]. The doped metal cation ions in the MoSe_2_ can enhance the electron transportation within the active materials during the HER process. Qian et al. [29] reported the production of Zn-doped MoSe_2_ nanosheets synthesized by a one-step hydrothermal method. When tested in 0.5 M H_2_SO_4_, the Zn-doped MoSe_2_ exhibited an overpotential of 231 mV at 10 mA cm^−2^, lower than that of the undoped pure MoSe_2_. Additionally, carbon matrices have excellent electric conductivity, mechanical flexibility and stable physical and chemical properties. When compositing carbon with pure MoSe_2_, the overall electron conductivity of the composite could be improved. Moreover, the protective carbonaceous layers can avoid direct contact between the MoSe_2_ and the electrolyte, therefore alleviating the structural collapse and surface side reactions of active materials. For instance, Xu et al. [30] fabricated core–shell MoSe_2_/C nanospheres by a hydrothermal method. Glucose-derived amorphous carbon could effectively enhance the conductivity and reduce the layers thickness of the MoSe_2_, leading to a low current density of 57.5 mA cm^−2^ at an overpotential of 200 mV. Liu et al. [31] synthesized flake MoSe_2_/C composites by a simple solid phase method. The carbon acting as a conductive matrix in the MoSe_2_/C composite material can not only facilitate electron transfer within the composites but also improve its structural stability and hinder the aggregation of MoSe_2_ nanosheets. Ren et al. [32] reported the fabrication of C-@MoSe_2_ composite by a sol–gel method, showing a potential of 170 mV at 10 mA cm^−2^ and a slope of 72 mV dec^−1^.

Heteroatom doping in carbon materials can create many defects, expand the specific surface area, enlarge the active sites and improve the electrical conductivity, which is beneficial to the enhancement of HER performance [33]. Furthermore, the rational design of micro/nanostructure such as a porous structure, hierarchical structure or core–shell structure can also improve the specific surface area, shorten the ion and electron transport pathway and facilitate the electrolyte penetration. Importantly, the continuous three-dimensional (3D) carbonaceous network ensures the rapid transmission of electrons in all directions to improve the overall electrical conductivity of the composites. Moreover, the 3D conductive network acts as protective matrix that can buffer the volume changes in the inner active materials and guarantee excellent structural integrity of the composite material. Therefore, constructing a continuous 3D carbonaceous network could be a promising method to improve the HER performance of the MoSe_2_-based materials [34].

Herein, the honeycomb-like MoSe_2_/NC composite was prepared by a freeze-drying method followed by an in situ selenization process. The ion and electron conductivity and structural stability of the MoSe_2_/NC have been effectively improved by the continuous honeycomb-like carbonaceous network. Moreover, the nitrogen-doped carbon matrix has numerous defects and a high specific surface area, further increasing the electrochemical performance of the obtained samples. When tested at 1 M KOH, the MoSe_2_/NC delivers overpotentials of 153 mV and 180 mV for HER and OER at 10 mA cm^−2^, respectively. Moreover, the MoSe_2_/NC‖MoSe_2_/NC cell requires a potential of only 1.5 V to reach the current density of 10 mA cm^−2^ for overall water splitting.

## 2. Results and Discussion

**Figure 1:**
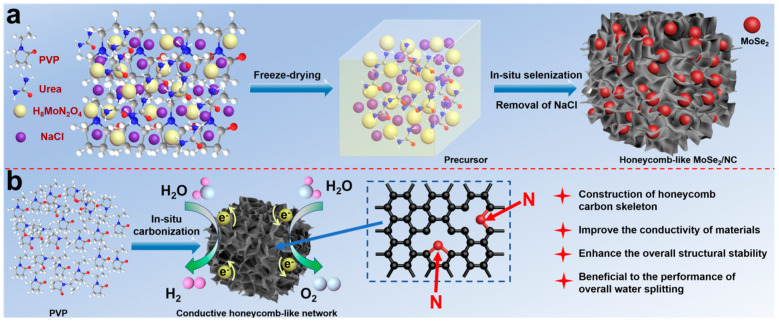
(**a**) Schematic illustration of the fabrication process of the honeycomb-like MoSe_2_/NC composite; (**b**) The advantages of MoSe_2_/NC.

The unique construction of the honeycomb-like carbon skeleton-encapsulated MoSe_2_/NC was synthesized by a facile freeze-drying method combined with the in situ selenization process. As shown in Figure 1a, the ammonium molybdate, PVP, urea and NaCl were mixed in a one-step freeze-drying process to form a polymer framework precursor. After that, the precursor underwent a facile selenization process at 600 °C in an Ar atmosphere. During this stage, the polymer framework was transformed into an N-doped carbonaceous network with the participation of urea. Significantly, nitrogen atoms replace carbon atoms, creating lattice defects (Figure 1b), whereas the inner ammonium molybdate was in situ converted into a MoSe_2_ phase. Furthermore, abundant orderly macropores with honeycomb-like features can be constructed within the composite by just removing the NaCl. The unique honeycomb-like conductive network could be helpful to the ion and electron transportation, electrolyte penetration and overall structural stability, which is beneficial to the performance of overall water splitting.

**Figure 2:**
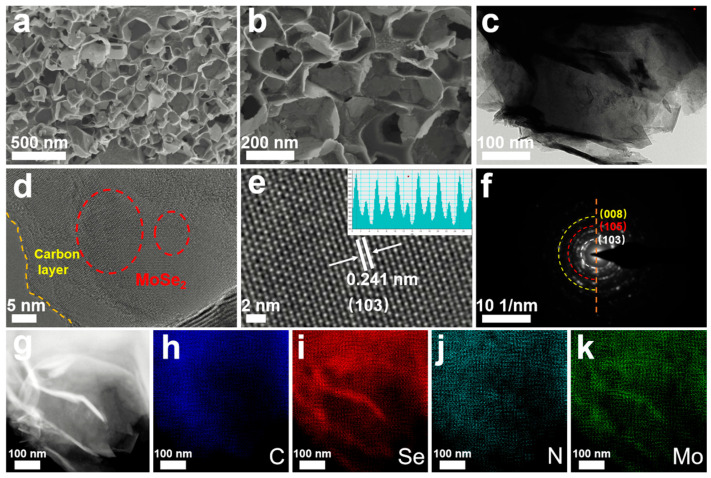
The morphologies and interior structures of the obtained samples. (**a**,**b**) SEM images. (**c**) TEM images. (**d**,**e**) HRTEM images. (**f**) The selected-area electron diffraction (SAED) patterns. (**g**–**k**) High-angle annular dark field scanning transmission electron microscopy (HAADF-STEM) and elemental mapping images of the MoSe_2_/NC composite.

The morphologies and interior structures of the MoSe_2_/NC, the MoSe_2_/C and the commercial MoSe_2_ were analyzed by SEM and TEM measurements. According to Figure 2a, the MoSe_2_/NC shows a uniform interconnected porous structure, and no obvious structural collapse is detected. A higher magnification image (Figure 2b) reveals that the porous structure is constructed by nanoplates with a diameter of around 100–200 nm. Moreover, the nanoplate seems to have a rough surface, which could be attributed to the decomposition of polymer and urea. The MoSe_2_/C (Figure S1a,b) appears to have structural collapse after the selenide process, demonstrating that urea could be helpful in the improvement of structural stability for the sample during the heat treatment process. Figure S2a,b show the SEM images of the commercial MoSe_2_ particles with a diameter of around 8 μm. More detailed information of the obtained samples was investigated by TEM measurement. As shown in Figure 2c, the MoSe_2_/NC composite is constructed by numerous nanoplates which display multi-layered and ultrathin features. Therefore, the porous structure of the MoSe_2_/NC is mainly due to the orderly stacking of hierarchical nanoplates. Figure 2d shows a high-resolution TEM image of the MoSe_2_/NC composite. Abundant lattice fringes can be observed in the composite, confirming that the active materials are well embedded into the honeycomb-like carbonaceous network. The magnified TEM image (Figure 2e) further recognizes that the lattice spacing is 0.241 nm, belonging to the (103) space of the standard MoSe_2_ and demonstrating that the nanocrystalline in the carbon skeleton is ascribed to MoSe_2_. Figure 2f shows the selected-area electron diffraction (SAED) patterns of the MoSe_2_/NC, and the result confirms the existence of the MoSe_2_ phase. As shown in Figure 2g–k, the elemental mapping results again verify the existence of Mo, Se, C and N elements in the composite. The red and green areas in Figure 2i,k suggest that the MoSe_2_ nanocrystalline is uniformly distributed in the honeycomb-like carbon network.

**Figure 3:**
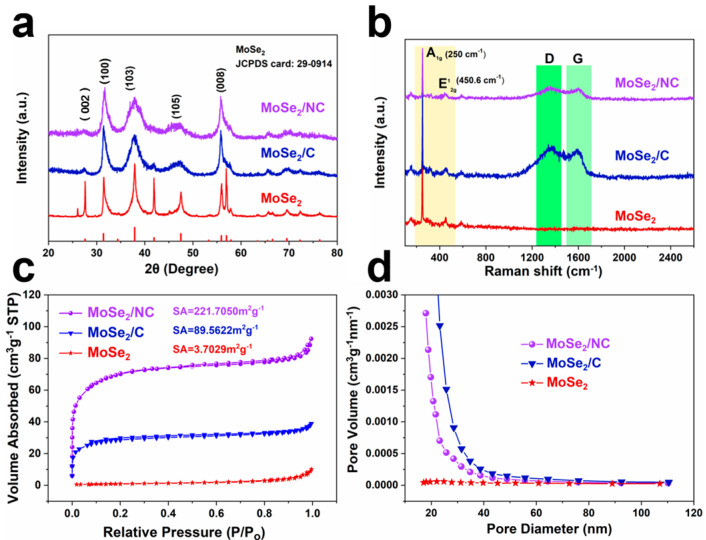
(**a**) XRD patterns. (**b**) Raman spectra. (**c**) Adsorption and desorption isotherm curves. (**d**) Pore size distribution curves of the MoSe_2_/NC, the MoSe_2_/C, and the commercial MoSe_2_ composites.

Figure 3a shows the XRD patterns of the samples after the selenization process. For the MoSe_2_/NC, obvious diffraction peaks can be detected at around 31.7°, 37.9°, 47.1° and 55.7°, corresponding to the planes of (100), (103), (105) and (108) for the standard MoSe_2_ (JCPDS 29-0914), respectively. No other residual peaks are detected, indicating the high purity of the MoSe_2_/NC composite, consistent with the TEM result as well. In the absence of urea, the product (MoSe_2_/C) shows similar diffraction peaks to the MoSe_2_/NC, which confirms that the MoSe_2_ phase can be easily synthesized by the fabrication strategy in our work. In addition, the commercial MoSe_2_ was characterized, and the diffraction peaks correspond well to the standard MoSe_2_ phase (JCPDS 29-0914). Compared with commercial MoSe_2_, the MoSe_2_/NC and the MoSe_2_/C display broader diffraction peaks and weaker peak intensities. These peak characteristics represent the lower crystallinity of the MoSe_2_/NC and the MoSe_2_/C, which could be ascribed to the abundant amorphous carbonaceous matrix in the composite. Raman measurements were carried out to further investigate the bonding information and properties of the carbon in the obtained samples. As shown in Figure 3b, three samples show similar peaks between 200 cm^−1^ and 560 cm^−1^, attributed to the characteristic peaks of the MoSe_2_ [26]. Moreover, two obvious peaks located at about 1360.07 cm^−1^ and 1584.83 cm^−1^ can be detected for both the MoSe_2_/NC and the MoSe_2_/C, which can be ascribed to the amorphous carbon (D band) and graphitic carbon (G band), respectively [30]. The peak intensity ratio of the D band and the G band (ID/IG) can reflect the graphitic degree of the composite. The ID/IG values of the MoSe_2_/NC and the MoSe_2_/C are 1.06 and 1.09, respectively, indicating that carbon in the MoSe_2_/NC and the MoSe_2_/C is mainly dominated by graphitized carbon. The porous structure of the three samples was further investigated by the N_2_ adsorption and desorption measurements. The XPS spectra of Mo3d, N1s and C1s (Figure S5) demonstrate that carbon and nitrogen have been successfully doped in the MoSe_2_/NC, which could effectively enhance the catalytic activity of the obtained catalysts. Figure 3c shows the N_2_ adsorption and desorption isotherm curves of the MoSe_2_/NC, the MoSe_2_/C and the MoSe_2_. The three curves can be assigned to H_2_ type (Type II Isotherm: S-type isotherm), indicating a mesoporous structure. Based on the Brunauer–Emmett–Teller (BET) method, the specific surface areas of the MoSe_2_/NC, the MoSe_2_/C, and the commercial MoSe_2_ were calculated to be 221.70, 89.56 and 3.70 m^2^g^−1^, respectively. A large specific area usually has abundant active sites and is beneficial to the improvement of ion and electron diffusion rates and electrolyte penetration. Figure 3d shows the pore size distributions of the samples. The pore size is mainly distributed between 20 and 80 nm, confirming the mesoporous structure of the three samples.

**Figure 4:**
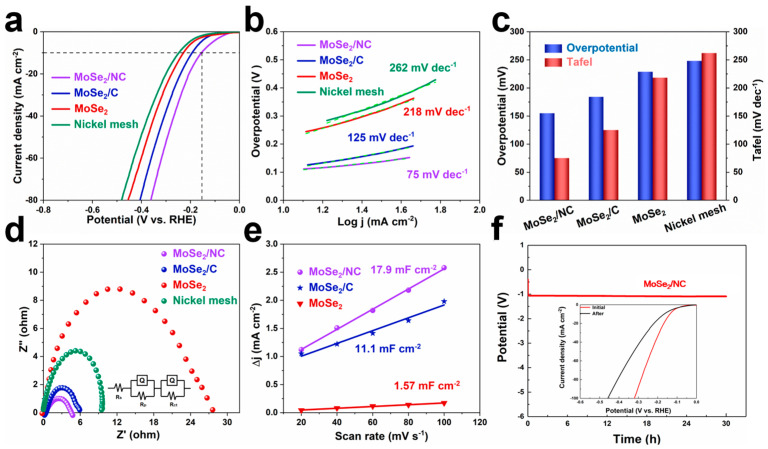
Electrochemical properties of the MoSe_2_/NC, the MoSe_2_/C, the commercial MoSe_2_ and the pure nickel foam for HER. (**a**) LSV curves. (**b**) Corresponding Tafel plots. (**c**) Comparison diagram of LSV and Tafel. (**d**) Nyquist plots. (**e**) Calculated Cdl of the obtained samples in 1 M KOH aqueous solution. (**f**) Electrochemical stability of the MoSe_2_/NC electrode at different current densities for 30 h (Inset: LSV curves of the MoSe_2_/NC before and after stability measurement).

The obtained samples were coated onto nickel foam to evaluate the HER performance by testing in 1 M KOH aqueous solution. As shown in Figure 4a, the MoSe_2_/NC electrode displays the best HER performance with an overpotential of 153 mV at 10 mA cm^−2^ and a low onset overpotential of only 50 mV. In comparison, the MoSe_2_/C and the commercial MoSe_2_ show overpotentials of 184 mV and 219 mV at 10 mA cm^−2^, respectively, higher than that of the MoSe_2_/NC. The pure nickel foam without active materials was also tested and exhibits relatively poor electrochemical performance (an overpotential of 284 mV at 10 mA cm^−2^), consistent with previous studies. Tafel plots were constructed from the polarization curves to elucidate the HER mechanism. The most advanced and fastest HER process should be determined by the Tafel reaction process of hydrogen recombination, which implies that a smaller Tafel slope dictates a faster HER process. Consistent with the LSV results, the Tafel slopes were also optimized for the samples with the honeycomb-like carbon framework. As shown in Figure 4b, the Tafel slopes of the MoSe_2_/NC and the MoSe_2_/C were determined to be 75 mV dec^−1^ and 125 mV dec^−1^, respectively, lower than that of the commercial MoSe_2_ (218 dec^−1^) and the pure metal matrix (262 mV dec^−1^). Therefore, the values indicate that the HER process can be inferred as the Volmer−Heyrovsky mechanism on the obtained electrodes [34]. The enhanced HER activity of the MoSe_2_/NC was further evaluated and compared in Figure 4c. The result demonstrates that the MoSe_2_/NC electrode exhibits the best overpotential and Tafel slope, which could be attributed to the abundant active sites and stable structure induced by the honeycomb-like carbonaceous network derived from the polymer by freeze-drying and pyrolysis. Meanwhile, the robust carbon skeleton can prevent the aggregation of the inner MoSe_2_ nanocrystalline during the electrochemical process [32].

To investigate the electrode kinetics during the HER process, the charge transfer resistance (R_ct_) at −1.27 V (vs. RHE) of the obtained samples was measured by an electrochemical impedance spectroscopy (EIS) technique. As shown in Figure 4d, all the curves consist of semicircular shapes. The Nyquist plots indicate that the MoSe_2_/NC has the smallest R_ct_ value (about 5 Ω), whereas those of the MoSe_2_/C, the commercial MoSe_2_ and the nickel foam are 6, 10 and 27 Ω, respectively. This result demonstrates that the MoSe_2_/NC has the fastest charge transfer and more favorable reaction kinetics for HER catalysis. Furthermore, the specific surface area (ECSA) of the obtained samples was evaluated by CV tests at different scanning rates of 20, 40, 60, 80 and 100 mV s^−1^ (Figure S3) to reveal the mechanism of the best HER performance for the MoSe_2_/NC electrode. The double-layer capacity (C_dl_) (Figure 4e) was calculated based on CV measurements to evaluate the ECSA values of the three molybdenum-based composites. The MoSe_2_/NC possessed a C_dl_ value of 17.9 mF cm^−2^, larger than that of the MoSe_2_/C (11.1 mF cm^−2^) and the commercial MoSe_2_ (1.57 mF cm^−2^). The highest ECSA of the MoSe_2_/NC indicates that the porous carbon framework leads to abundant active sites for the electrochemical reactions. The higher ECSA value of the MoSe_2_/NC compared to the MoSe_2_/C could be ascribed to the extra structural defects caused by the N-doped carbon and uniform honeycomb-like structure, consistent with previous studies [34]. The superior electrochemical performance of the MoSe_2_/NC was confirmed by the stability performance test. As shown in Figure 4f, the MoSe_2_/NC showed good stability at 10 mA cm^−2^. The LSV curves of this test (seen in the Figure 4f inset) also demonstrate the excellent durability of the MoSe_2_/NC. The LSV curves of the initial and after 30 h tests (inset in Figure 4f) also verify the excellent durability of the MoSe_2_/NC. The improved HER activity of the MoSe_2_/NC may be attributed to its unique honeycomb-like network structure and N-doped carbon, which results in robust structural flexibility and fast ion and electron transportation.

**Figure 5:**
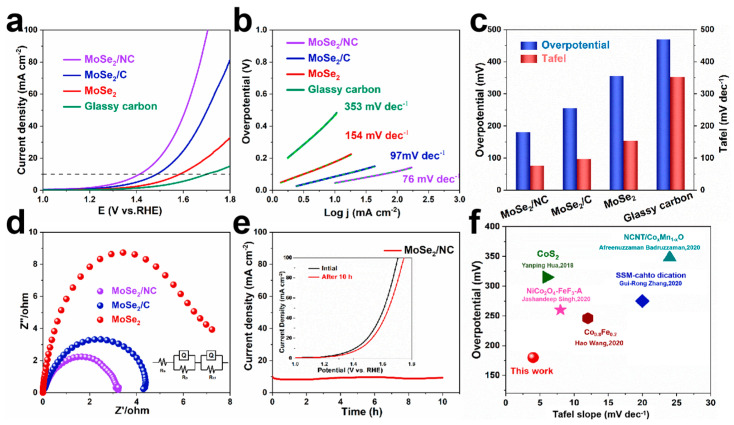
Electrochemical properties of the MoSe_2_/NC, the MoSe_2_/C and the MoSe_2_ for OER. (**a**) LSV curves. (**b**) Corresponding Tafel plots. (**c**) Comparison diagram of LSV and Tafel. (**d**) Nyquist plots of the obtained three samples. (**e**) Electrochemical stability of the MoSe_2_/NC after 10 h test at constant point. (Inset: LSV curves of the MoSe_2_/NC before and after stability tests.) (**f**) Comparison of the overpotentials between our work and previous studies [36,37,38,39,40].

The OER catalytic activities of the prepared samples were further evaluated by LSV and EIS measurements in a 1 M KOH solution at a scan rate of 5 mV s^−1^. As shown in Figure 5a, the MoSe_2_/NC delivers an overpotential of only 180 mV at 10 mA cm^−2^, much lower than those of the MoSe_2_/C (255 mV) and the MoSe_2_ (355 mV). Moreover, the MoSe_2_/NC possesses the best overpotential even at a high current density of 50 mA cm^−2^. In addition, the MoSe_2_/NC displays the smallest Tafel slope of 76 mV dec^−1^ among the obtained samples, such as the MoSe_2_/C (97 mV dec^−1^) and the commercial MoSe_2_ (154 mV dec^−1^) (Figure 5b). Figure 5c shows the overpotentials and Tafel slopes of the obtained three samples and glassy carbon electrode, demonstrating the superior catalytic activity of the MoSe_2_/NC toward OER. The measurement of current response vs. the operation time was carried out to investigate the stability of the MoSe_2_/NC. The fast electron and ion transportation and favorable structural integrity derived from the robust honeycomb-like carbonaceous network could enhance the OER catalytic activity of the MoSe_2_/NC. To confirm the above conjecture, EIS curves and stability testing of the samples were carried out. Figure 5d shows Nyquist plots for the MoSe_2_/NC, the MoSe_2_/C and the commercial MoSe_2_ in 1 M KOH electrolyte at a potential of 1.64 V vs. RHE. The solution resistance (R_s_) and the charge transfer resistance (R_ct_) are related to the size of the semicircle in the low- and high-frequency regions. The EIS value of the MoSe_2_/NC is about 3.2 Ω, smaller than that of the MoSe_2_/C (4.5 Ω) and the commercial MoSe_2_ (7.8 Ω). As shown in Figure 5e, the electrode still has excellent current density retention after durability testing for 10 h. In addition, the LSV curves of the initial and after long-term stability measurements (inset in Figure 5e) were tested, and the nearly overlapping curves indicate the superior electrochemical performance. The morphology characterization of the MoSe_2_/NC after the stability test (Figure S4a,b) shows that the honeycomb-like structure can be well maintained during the OER process, verifying the robust stability of the porous carbon network. Compared with previous studies, the MoSe_2_/NC obtained in our work exhibits great potential as an advanced catalyst for OER (Figure 5f) [35].

**Figure 6:**
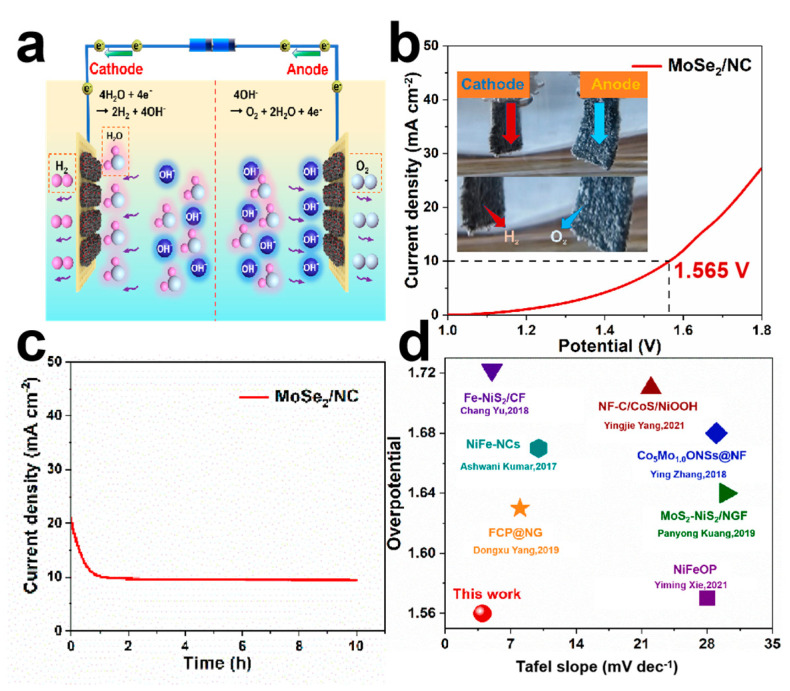
The electrochemical performance of the MoSe_2_/NC‖MoSe_2_/NC cell during overall water splitting. (**a**) Schematic diagram of the MoSe_2_/NC‖MoSe_2_/NC electrolyzer. (**b**) LSV curves of the MoSe_2_/NC‖MoSe_2_/NC cell. (Inset: a camera picture of the electrode during water splitting.) (**c**) Stability test of the MoSe_2_/NC‖MoSe_2_/NC cell. (**d**) The comparison of the cell voltage for our electrolyzer with previous reports [43,44,45,46,47,48,49].

The remarkable HER and OER performance of the MoSe_2_/NC motivates us to further explore its practical performance as both the anode and the cathode for overall water splitting by constructing a two-electrode electrolyzer in alkaline conditions (schematically represented in Figure 6a). Figure 6b shows that the MoSe_2_/NC‖MoSe_2_/NC cell requires a potential of 1.5 V to achieve the current density of 10 mA cm^−2^ during overall water splitting, which is comparable to the commercial Pt/C‖RuO_2_ cell. Figure 6c further shows the durability performance of the MoSe_2_/NC at 10 mA cm^−2^ in a 1 M KOH solution for long-term operation [41]. The current density can still be maintained at 9.1 mA cm^−2^ after 10 h with a retention of 91%, suggesting excellent durability of the MoSe_2_/NC electrode during overall water splitting after long-term operation. The corresponding SEM images after testing (Figure S6) confirm the considerable structural stability during the catalytic process. As compared with previously developed catalysts, the MoSe_2_/NC exhibits outstanding electrochemical performance, indicating that it is one of the best potential bifunctional catalysts for overall water splitting (Figure 6d) [42].

## 3. Experimental Section

### 3.1. Synthesis of the MoSe_2_/NC Composite

The MoSe_2_/NC was fabricated by the freeze-drying method combined with an in situ selenization process. Typically, 10 g polyvinylpyrrolidone (PVP, Mw = 1,300,000) was first dissolved in 100 mL deionized water. Then, 5.6 g ammonium molybdate, 5 g urea and 10 g NaCl were also added to the above solution and vigorously stirred for 2 h to obtain a transparent solution. After that, the transparent solution was treated using a freeze-drying method for 24 h. Finally, the obtained precursor (1 g) was mixed with selenium powder (2 g), and then the mixture was annealed at 600 °C for 4 h under an Ar atmosphere with a temperature rate of 3 °C min^−1^. After cooling down naturally, the samples were washed with water to remove the NaCl, and the final product was named MoSe_2_/NC. For comparison purposes, the MoSe_2_/C was also prepared by a similar method without the addition of urea. The commercial MoSe_2_ particles were purchased from Sigma-Aldrich (Shanghai, China).

### 3.2. Structural Characterization

X-ray diffraction (XRD) measurements were performed on a D/Max 2700 X-ray diffractometer with Cu-Kα radiation. The structure and morphology of the prepared samples were characterized by field-emission scanning electron microscopy (FESEM FEI Nova Nano SEM 230, Beijing, China) and transmission electron microscopy (TEM, JEOL-JEM-2100F, Shanghai, China). Raman spectroscopy was obtained on a Renishaw 1000. The specific surface area and pore size distribution of the MoSe_2_/NC were characterized with a surface area detecting instrument by N_2_ physisorption (ASAP 2020 HD88).

### 3.3. Electrochemical Measurements

All electrochemical tests were performed on a CHI 660e electrochemical workstation using a standard three-electrode test. During the HER test, the active materials and Polyvinylidene fluoride (PVDF) with a mass ratio of 9:1 were mixed and dispersed into N-methyl pyrrolidone (NMP) to form a slurry. Then, the slurry was coated onto nickel mesh foam (a size of 1 cm × 2 cm) and dried in a vacuum at 60 °C overnight. A calomel electrode and carbon rod were used as the reference electrode and the counter electrode, respectively. During the OER test, active material (2 g), nafion solution (40 μL), absolute ethanol (120 μL) and deionized water (840 μL) were mixed together and subjected to ultrasound for 30 min to obtain the uniform slurry. After that, 5 μL dispersion was dropped onto the glassy carbon electrode and dried overnight at room temperature for the OER test. The entire test was carried out in 1 M KOH aqueous solution. Linear sweep voltammetry (LSV) was measured at a scan rate of 5 mV s^−1^. The electrochemical impedance spectroscopy (EIS) was investigated with a frequency range of 105-10-2 Hz (vs. RHE) using 5 mV amplitude. The electrochemical surface area (ECSA) was evaluated by CV tests under different scanning rates (10, 20, 40, 60, 80, 100 mV s^−1^) with the voltage range of 0–0.10 V (versus RHE).

## 4. Conclusions

In summary, the honeycomb-like N-doped carbon network-encapsulated MoSe_2_/NC composite was fabricated by a facile freeze-drying method combined with an in situ selenization strategy. The rationally designed 3D hierarchical and porous structure endows abundant active sites, fast ion and electron transportation channels and robust structural flexibility. Moreover, N-doped carbon provides many defects, further enhancing the conductivity of the composite. The as-prepared MoSe_2_/NC electrode exhibits favorable catalytic activity and long-term durability for both HER and OER operation in an alkaline electrolyte. When utilized as a bifunctional electrode for overall water splitting, the MoSe_2_/NC‖MoSe_2_/NC cell requires a potential of only 1.5 V to reach the current density of 10 mA cm^−2^. The cell also delivers superior durability (current density retention of 91% after 10 h), suggesting great potential as a bifunctional electrocatalyst for overall water splitting. This work provides insight into developing a facile and scalable preparation strategy to fabricate advanced bifunctional electrodes for highly efficient hydrogen evolution.

## Data Availability

The original contributions presented in the study are included in the article/Supplementary Materials, further inquiries can be directed to the corresponding authors.

## Supplementary Materials

The following supporting information can be downloaded at https://www.mdpi.com/article/10.3390/molecules29163877/s1, Figure S1: The SEM images (a–c) of the MoSe_2_/C; Figure S2: The SEM images (a,b) of the commercial MoSe_2_ particles; Figure S3: CV curves of the MoSe_2_/NC (a), MoSe_2_/C (b) and commercial MoSe_2_ (c) at different scanning rates of 20, 40, 60, 80, 100 mV s^−1^; Figure S4: The SEM images (a,b) of the MoSe_2_/NC; Figure S5: The XPS spectra of the MoSe_2_/NC (a–d); Figure S6: The SEM images of the MoSe_2_/NC after testing for 10 h; Table S1: The comparison of catalytic performance between our work and previous reports. Refs. [43,44,45,46,47,48,49] are cited in the Supplementary Materials.
