# Supplementary material for: Rational Construction of Honeycomb-like Carbon Network-Encapsulated MoSe2 Nanocrystals as Bifunctional Catalysts for Highly Efficient Water Splitting
Source: Molecules. 2024 Aug 16;29(16):3877. doi: 10.3390/molecules29163877 (PMC11357002; doi:10.3390/molecules29163877)
Supplement: Supplementary file 1 [file molecules-29-03877-s001.zip › molecules-3144387-supplementary.pdf]

Supporting Information

Rational Construction of Honeycomb-Like Carbon Network-Encapsulated MoSe2 Nanocrystals as Bifunctional Catalysts for Highly Efficient Water Splitting

Changjie Ou [1], Zhongkai Huang [1], Xiaoyu Yan [1], Xiangzhong Kong [1,2,*], Xi Chen [2], Shi Li [1], Lihua Wang [1,2] and Zhongmin Wan [1,2,*]

[1] College of Mechanical Engineering, Hunan Institute of Science and Technology, Yueyang 414006, China; ouchangjie06@163.com (C.O.); 822311110535@vip.hnist.edu.cn (Z.H.); hunanligong4066@163.com (X.Y.); li_shi@csu.edu.cn (S.L.); hnlgwlh@163.com (L.W.)

[2] School of Energy and Electrical Engineering, Hunan Institute of Science and Technology, Yueyang 414006, China; xichen2013@hnu.edu.cn

* Correspondence: 12018024@hnist.edu.cn (X.K.); 12003021@hnist.edu.cn (Z.W.)

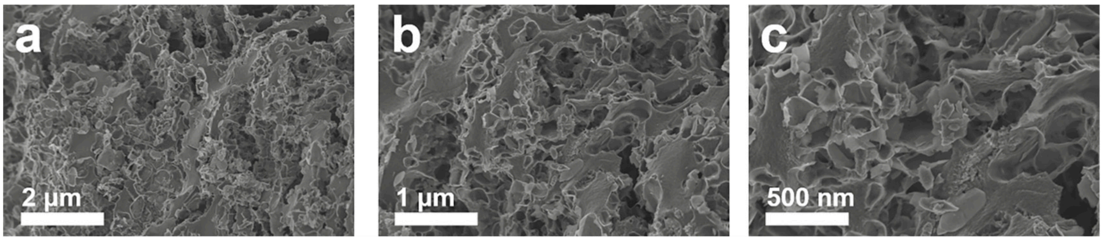

Figure S1. The SEM images (a–c) of the $MoSe_2$/C.

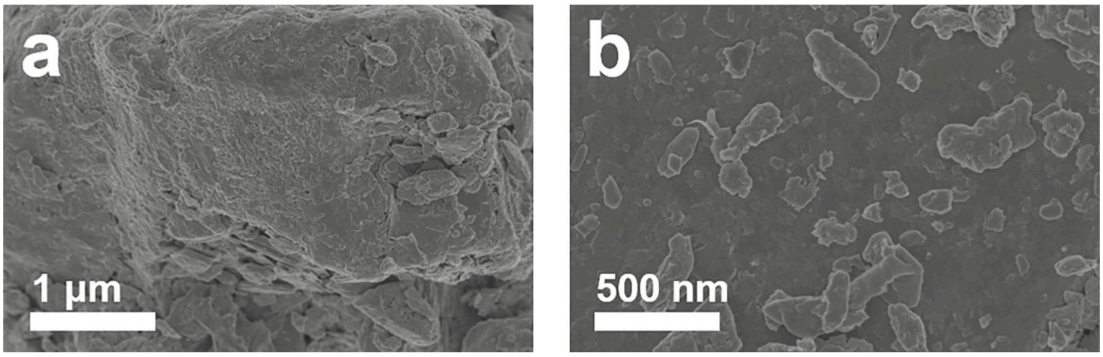

Figure S2. The SEM images (a, b) of the commercial $MoSe_2$ particles.

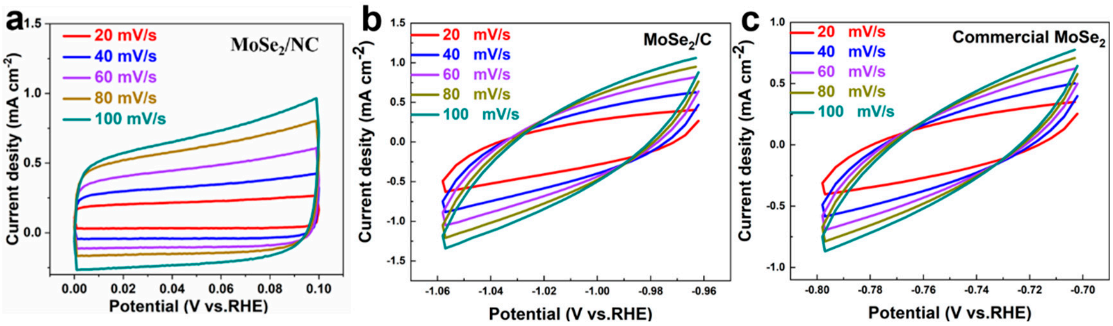

Figure S3. CV curves of the $MoSe_2$/NC (a), $MoSe_2$/C (b) and commercial $MoSe_2$ (c) at different scanning rates of 20, 40, 60, 80, 100 mV s^{-1}.

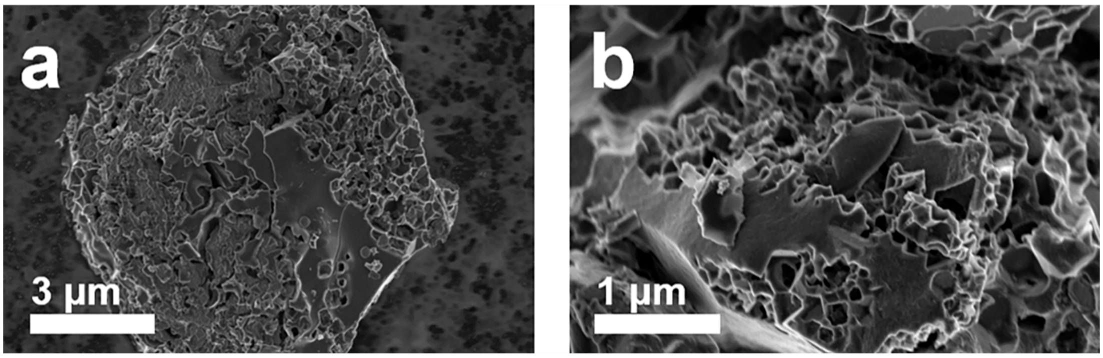

Figure S4. The SEM images (a, b) of the $MoSe_2$/NC.

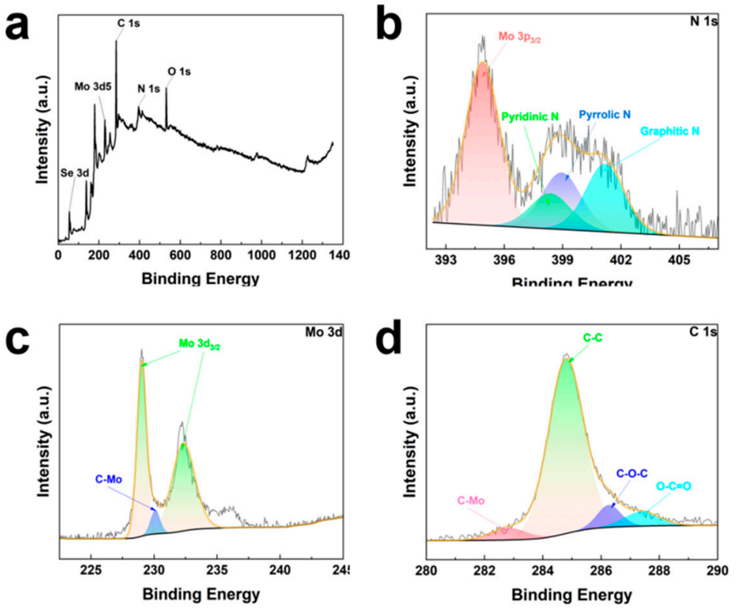

Figure S5. The XPS spectra of the $MoSe_2$/NC (a–d).

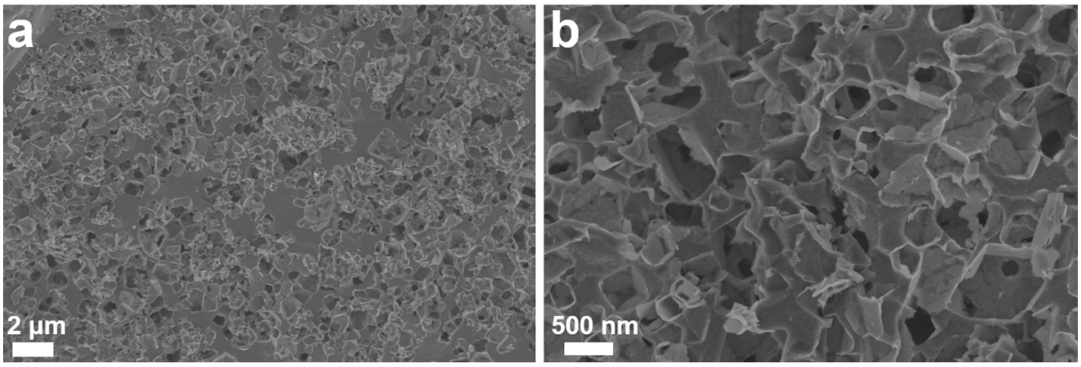

Figure S6. The SEM images (a,b) of the $MoSe_2$/NC after testing for 10 h.

Table S1. The comparison of catalytic performance between our work and previous reports.

Anode Materials	HER Overpotential (mV) (10 mA cm^{-2})	HER Tafel slope (mV dec^{-1})	OER Overpotential (mV) (10 mA cm^{-2})	OER Tafel slope (mV dec^{-1})	Ref.
$MoSe_2$/NC	153	75	180	76	our work
NiFeOP	153	105.25	217	62.7	[1]
FCP@NG	187	76	269	58	[2]
P-Ru/C	31	105	-	-	[3]
MoS_2-NiS_2/NGF	172	70	370	-	[4]
NiFe-NCs	271	48	197	130	[5]
Fe-NiS_2/CF	-	-	243	88	[6]
Co5MoONSs@NF	173	190.1	270	54.4	[7]

References

1. Xie, Y.; Zhao, B.; Tang, K.; Qin, W.; Tan, C.; Yao, J.; Li, Y.; Jiang, L.; Wang, X.; Sun, Y., In-situ phase transition induced nanoheterostructure for overall water splitting. *Chemical Engineering Journal* 2021, 409, 128156.
2. Yang, D.; Hou, W.; Lu, Y.; Zhang, W.; Chen, Y., Scalable synthesis of self-assembled bimetallic phosphide/N-doped graphene nanoflakes as an efficient electrocatalyst for overall water splitting. *Nanoscale* 2019, 11, (27), 12837-12845.
3. Yang, Y.; Yu, Y.; Li, J.; Chen, Q.; Du, Y.; Rao, P.; Li, R.; Jia, C.; Kang, Z.; Deng, P.; Shen, Y.; Tian, X., Engineering Ruthenium-Based Electrocatalysts for Effective Hydrogen Evolution Reaction. *Nanomicro Lett* 2021, 13, 160-169.
4. Kuang, P.; He, M.; Zou, H.; Yu, J.; Fan, K., 0D/3D MoS2-NiS2/N-doped graphene foam composite for efficient overall water splitting. *Applied Catalysis B: Environmental* 2019, 254, 15-25.
5. Kumar, A.; Bhattacharyya, S., Porous NiFe-Oxide Nanocubes as Bifunctional Electrocatalysts for Efficient Water-Splitting. *ACS Appl Mater Interfaces* 2017, 9, (48), 41906-41915.
6. Yu, C.; Huang, H.; Zhou, S.; Han, X.; Zhao, C.; Yang, J.; Li, S.; Guo, W.; An, B.; Zhao, J.; Qiu, J., An electrocatalyst with anti-oxidized capability for overall water splitting. *Nano Research* 2018, 11, (6), 3411-3418.
7. Zhang, Y.; Shao, Q.; Long, S.; Huang, X., Cobalt-molybdenum nanosheet arrays as highly efficient and stable earth-abundant electrocatalysts for overall water splitting. *Nano Energy* 2018, 45, 448-455.
